# Antioxidant Action of Dinitrosyl Iron Complexes in Model Systems Containing Cytochrome *c* and Organic Hydroperoxides

**DOI:** 10.3390/molecules30204110

**Published:** 2025-10-16

**Authors:** Olga V. Kosmachevskaya, Elvira I. Nasybullina, Konstantin B. Shumaev, Alexey F. Topunov

**Affiliations:** Bach Institute of Biochemistry, Research Center of Biotechnology, Russian Academy of Sciences, Moscow 119071, Russia; lvirus198709@rambler.ru (E.I.N.); tomorov@mail.ru (K.B.S.); aftopunov@yandex.ru (A.F.T.)

**Keywords:** cytochrome *c*, dinitrosyl iron complexes, nitric oxide, free radicals, organic peroxides, prooxidant and antioxidant effect

## Abstract

The antioxidant/prooxidant effects of dinitrosyl iron complexes (DNICs), physiological donors of nitric oxide (NO^•^), are studied in reaction systems modeling processes with cytochrome *c* occurring in mitochondria under oxidative stress and leading to apoptosis. Using luminol-dependent chemiluminescence, DNICs with glutathione and phosphate ligands were shown to decrease the level of prooxidants in a reaction system containing ferricytochrome *c* and cumene hydroperoxide. Electron paramagnetic resonance (EPR) spectroscopy revealed that glutathione DNICs (DNICs-GS) intercepted the free radicals formed during the interaction between cytochrome *c* and *tert*-butyl hydroperoxide. DNICs-GS were also shown to prevent the formation of oligomeric forms of cytochrome *c*, which were induced by organic hydroperoxides. Reduced glutathione was less effective as an antioxidant than DNICs-GS or could even occasionally exhibit the prooxidant properties. Ferricytochrome *c* also catalyzed the formation of DNICs-GS with nitroxyl anion (NO^−^) taking part.

## 1. Introduction

Cytochrome *c* is an essential component of a mitochondrial electron transport (respiratory) chain [1,2]. This protein also plays a crucial role in promoting apoptosis [3,4]. To initiate apoptosis, it is necessary to trigger the production of reactive oxygen species (ROS) by mitochondria, as well as the peroxidation of mitochondrial inner membrane lipids catalyzed by cytochrome *c*. The most effective is the complex of cardiolipin and cytochrome *c* [3,4,5]. In the presence of hydrogen peroxide (H_2_O_2_), cytochrome *c* causes free radical oxidation of phospholipids and unsaturated fatty acids [6], as well as the tyrosine residues of cytochrome itself [7]. Additionally, free radical intermediates are formed through cytochrome *c* reacting with cholesterol, *tert*-butyl, and cumene hydroperoxides [8,9,10,11].

Apart from contributing to ROS production, nitric oxide (NO^•^) participates in apoptosis regulation. Consequently, NO^•^ and its metabolites exhibit both prooxidant and antioxidant properties [12,13,14,15,16]. It has been shown that nitrosylation of the heme iron of ferro- and ferri- forms of cytochrome *c* inhibits the peroxidase activity of this protein and cardiolipin oxidation [17]. Meanwhile, ferricytocrome *c* reduces NO^•^ to the nitroxyl anion (NO^−^), in whose reaction with oxygen the strong oxidizing agent peroxynitrite (ONOO^−^) is formed [18]. Conversely, ONOO^−^ is formed when NO^•^ reacts with a superoxide anion radical (O_2_^•−^) [14,16]. Furthermore, ferricytochrome *c* is capable of oxidizing O_2_^•−^ to O_2_, which is considered as evidence in favor of the antioxidant properties of cytochrome *c* [19]. The combined effect of cytochrome *c* and NO^•^ on free radical oxidation processes in mitochondria is assumed to be highly ambivalent.

**Figure 1:**
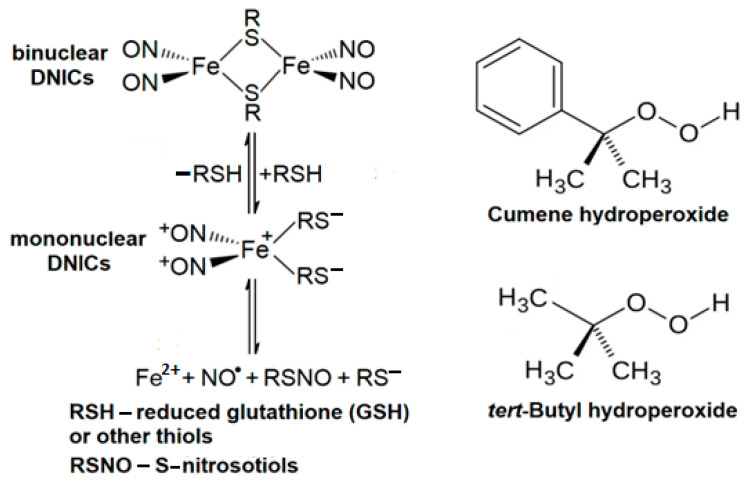
The formulae of iron dinitrosyl iron complexes (DNICs) and organic hydroperoxides (ROOH) used in the work.

NO^•^ interacting with non-heme iron results in the formation of dinitrosyl iron complexes (DNICs), having a wide range of biological activities [20,21,22,23]. In biological systems, DNICs include both NO^•^ and anionic ligands (thiol, phosphate, etc.), as well as histidine residues of proteins and peptides [21,22,24,25]. Thiol-containing DNICs exist in mononuclear and binuclear forms and can act as NO^•^ donors (Figure 1).

We have previously shown that DNICs with glutathione and carnosine ligands exhibit antioxidant and antiradical properties [22,24,26,27]. In addition, DNICs bound with hemoglobin protect this hemoprotein against oxidative modification by hydrogen peroxide and peroxynitrite [28,29,30,31]. It has also been shown that DNICs were formed in mitochondria with various NO^•^ sources and iron ions being involved [32,33,34]. The cytoprotective effect of DNICs may be connected with their ability to inhibit free radical oxidation [27,32,34]. However, under certain conditions, DNICs can exhibit cytotoxic properties [34,35], which may be attributed to the disruption of iron-containing enzymes [36,37].

It was clearly demonstrated [38] that cytochrome *c* can be nitrosylated in the presence of bivalent DNICs with thiosulfate ligands. However, the role of DNICs played in free radical peroxidation of biomolecules induced by cytochrome *c* has not yet been investigated. The aim of this study was therefore to examine the interaction between glutathione and phosphate DNICs (DNICs-GS and DNICs-PO_4_^−^, respectively) with various prooxidants formed during the cytochrome *c* reactions with organic hydroperoxides.

## 2. Results

### 2.1. The Effect of DNICs on Luminol-Dependent Chemiluminescence in Reaction Systems Containing Cytochrome c and Organic Hydroperoxides (Cumene Hydroperoxide and tert-Butyl Hydroperoxide)

Cumene hydroperoxide and *tert*-butyl hydroperoxide (*t*-BOOH) were used to study the metabolism of hydroperoxides, including their role in free radical oxidation of biomolecules [8,9,10,39,40,41,42].

**Figure 2:**
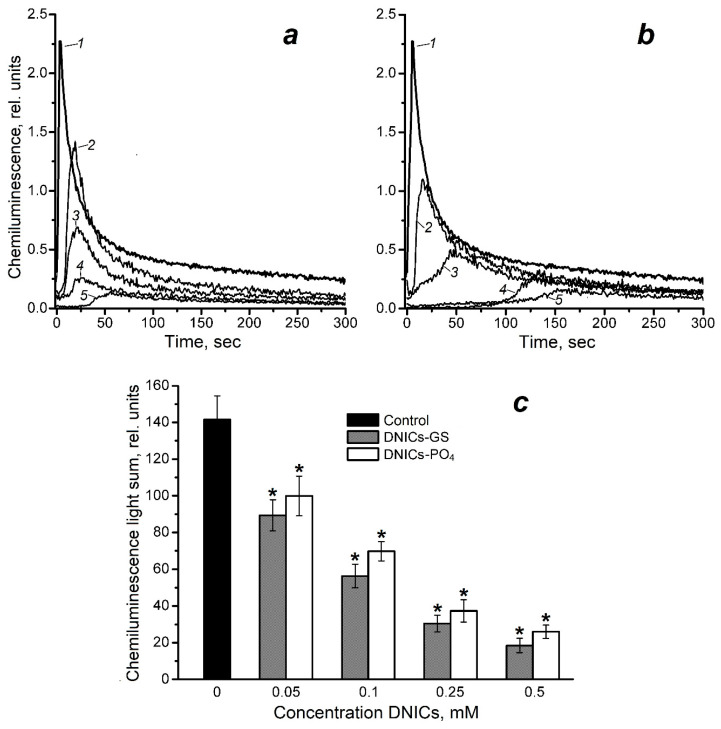
The effect of dinitrosyl iron complexes (DNICs) with glutathione (DNICs-GS) and phosphate (DNICs-PO_4_^−^ on the kinetics (**a**,**b**), and light sum (**c**) of luminol-dependent chemiluminescence in a system containing cytochrome *c* and cumene hydroperoxide (CumOOH). The composition of reaction mixture: 100 mM K, Na-phosphate buffer (pH 7.4), 0.6 mM ferricytochrome *c*, 0.6 mM CumOOH, 2 mM luminol and DNICs of various concentrations; 1—mixture without admixtures (the control), 2—(1) + 0.05 mM DNICs, 3—(1) + 0.1 mm DNICs, 4—(1) + 0.25 mM DNICs, 5—(1) + 0.5 mM DNICs. Groups that are significantly different from the control are marked *.

**Figure 3:**
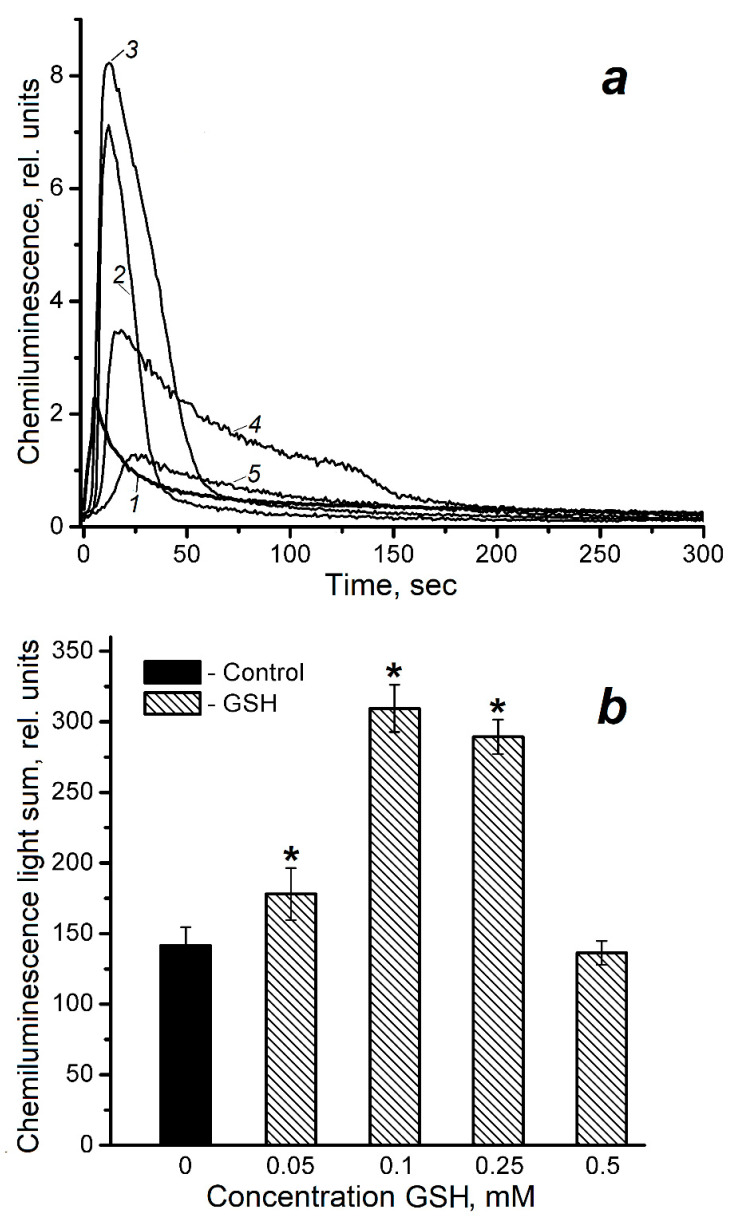
The effect of reduced glutathione (GSH) on the kinetics (**a**) and light sum (**b**) of luminol-dependent chemiluminescence in a system containing CumOOH and cytochrome *c*. Reaction mixture: 100 mM K, Na-phosphate buffer (pH 7.4), 0.6 mM ferricitochrome *c*, 0.6 mM CumOOH, 2 mM luminol and GSH of various concentrations. 1—mixture without admixtures (control), 2—(1) + 0.05 mM GSH, 3—(1) + 0.1 mM GSH, 4—(1) + 0.25 mM GSH, 5—(1) + 0.5 mM GSH. Groups that are significantly different from the control are marked *.

The effect of glutathione and phosphate DNICs on the level of free radicals and other prooxidants formed at the interaction of ferricytochrome *c* and cumene hydroperoxide (CumOOH) was studied by luminol-dependent chemiluminescence (Figure 2). The antioxidant effect of the studied compounds was evaluated with the following characteristics of the kinetic curve of chemiluminescence: light sum, luminescence intensity, and the latency period [24,25]. It should be noted that the chemiluminescence light sum is the most suitable method for quantifying the prooxidants content in this system (Figure 2 and Figure 3). DNICs were added to the reaction medium, which contained equimolar concentrations (0.6 mM) of ferricytochrome *c* and CumOOH, in a range of concentrations of 0.05–0.5 mM. As can be seen (Figure 2), these DNICs decreased the light sum of luminol-dependent chemiluminescence in a dose-dependent manner. DNICs-GS were found to reduce the yield of prooxidants by 1.6 and 4.6 times at concentrations of 0.05 and 0.25 mM, respectively. At 0.5 mM concentration, being close to the concentration of CumOOH in the reaction mixture, it reduced this yield by 7.6 times (Figure 2c). The antiradical effect of both DNIC types was comparable. A significant difference was observed only at 0.1 mM concentration, when DNICs-PO_4_^−^ and DNICs-GS reduced chemiluminescence light sum by 2 and 2.5 times, respectively.

Likewise, increasing the concentration of DNICs, decreased the maximal luminescence intensity and extended the latent period on chemiluminescence kinetic (Figure 2a,b). Meanwhile, the latent period was affected significantly more by DNICs-PO_4_^−^ than by DNICs-GS: at a concentration of 0.25 and 0.5 mM, the latent period for DNICs-PO_4_^−^ was 100 and 130 s, respectively, compared to 16 and 30 s for DNICs-GS.

Since DNICs-GS can degrade with the release of reduced glutathione (GSH), we studied the effect of this thiol on luminol-dependent chemiluminescence in the studied reaction system (Figure 3). Although GSH is considered to be an antioxidant, it almost doubled the chemiluminescence light sum in our experiments when cytochrome *c* interacted with CumOOH at concentrations of 0.1 and 0.25 mM. On the contrary, at concentrations of 0.05 mM and 0.5 mM, the GSH effect did not significantly differ from the control. However, GSH increased the latent period of the chemiluminescence curve, implicating the antioxidant effect.

Several studies have shown that the reactions of organic hydroperoxide with ferro- and ferricytochrome *c* (Cyt *c*-FeII and Cyt *c*-FeIII, respectively) produce alkoxyl (RO^•^) and peroxyl (ROO^•^) radicals (Reactions (1)–(5)) [9,10,40,42]:

ROOH + Cyt *c*-Fe^II^ ⟶ RO^•^ + Cyt *c*-Fe^III^ (1)

ROOH + Cyt *c*-Fe^III^ ⟶ RO^•^ + Cyt *c*-Fe^IV^=O (2)

ROOH + Cyt *c*-Fe^III^ ⟶ ROH + Cyt *c*^•+^-Fe^IV^=O (3)

ROOH + Cyt *c*^•+^-Fe^IV^=O ⟶ ROO^•^ + Cyt *c*-Fe^IV^=O (4)

ROO^•^ + ROO^•^ ⟶ 2 RO^•^ + O_2_ (5)

Of note, here we used ferricytochrome *c* preparation, which in the presence of GSH and DNICs-GS could be partially reduced to the ferrous form. In addition to free radicals, the highly oxidized forms of heme can also oxidize luminol [7,43]. These prooxidants include the oxoferryl (Cyt *c*-Fe^IV^=O) and perferryl (Cyt *c*^•+^-Fe^IV^=O) cytochrome *c* forms. It is likely that DNICs and/or their breakdown products can intercept free radicals and reduce the highly oxidized heme forms produced in Reactions (1)–(5). This assumption is consistent with the previously obtained results [24,25,26]. In our experiments, GSH may undergo one-electron oxidation to form glutathione thiyl radicals (GS^•^), which in turn transform into other free radical intermediates in the following Reactions (6)–(8) [44]:

GS^•^ + O_2_ → GSOO^•^ (6)

GS^•^ + GS¯→ GSSG^•^¯ (7)

GSSG^•^¯ + O_2_ → GSSG + O_2_^•^¯ (8)

The formation of GS^•^ and other free radicals in Reactions (6)–(8) may account for the controversial (non-uniform) effect of free GSH on luminol-dependent chemiluminescence in the cytochrome *c*/CumOOH reaction system.

**Figure 4:**
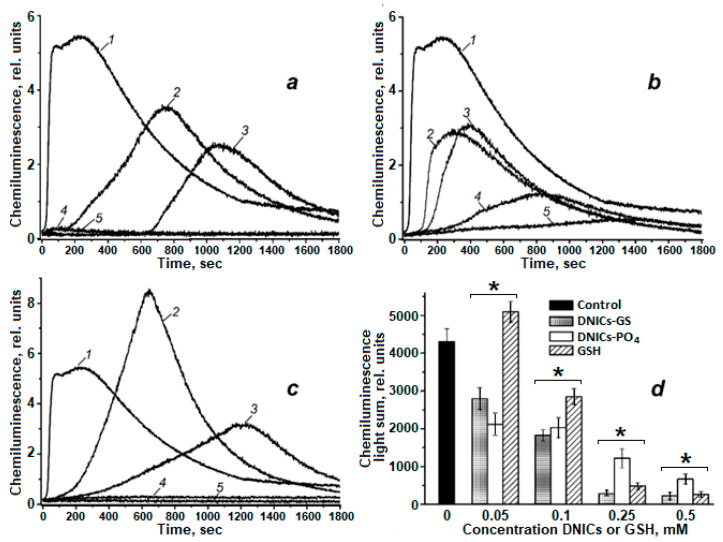
The effect of dinitrosyl iron complexes (DNICs-GS and DNICs-PO_4_^−^) or GSH on the kinetics (**a**–**c**) and light sum (**d**) of luminol-dependent chemiluminescence in a system containing cytochrome *c* and *tert*-butyl hydroperoxide (*t*-BOOH). The composition of reaction mixture: 100 mM K,Na-phosphate buffer (pH 7.4), 0.14 mM ferricytochrome *c*, 0.25 mM *t*-BOOH, 1 mM luminol and DNICs or GSH of various concentrations; 1—mixture without admixtures (the control), 2—(1) + 0.05 mM DNICs-GS/DNICs-PO_4_^−^/GSH, 3—(1) + 0.1 mm DNICs-GS/DNICs-PO_4_^−^/GSH, 4—(1) + 0.25 mM DNICs-GS/DNICs-PO_4_^−^/GSH, 5—(1) + 0.5 mM DNICs-GS/DNICs-PO_4_^−^/GSH. Groups that are significantly different from the control are marked *.

In a model system containing cytochrome *c* and *tert*-butyl hydroperoxide (*t*-BOOH), the yield of reactive products was significantly higher than in the system with cumene hydroperoxide (CumOOH) (Figure 4). Moreover, the kinetics of chemiluminescence in the *t*-BOOH system were more prolonged. Both types of DNICs, DNICs-GS and DNICs-PO_4_^−^, increased the latent period of the chemiluminescence curve and reduced both the total light emission (light sum) and maximum intensity of luminescence (Figure 4a–d). Particularly pronounced elongation of the latent period was observed under the action of DNICs-GS: at 0.05 mM DNICs-GS, the latent period was 165 s, and at 0.1 mM—660 s. In comparison, at the same concentrations of DNICs-PO_4_^−^, the latent period was 80 and 130 s, respectively.

The effect of DNICs on the light sum was also analyzed. At concentrations of 0.05 and 0.1 mM, DNICs-PO_4_^−^ reduced the light sum by approximately two-fold compared to the control (Figure 4d); increasing their concentration to 0.25 and 0.5 mM led to reductions in the light sum by 3.5- and 6.4-fold, respectively. DNICs-GS at 0.05 and 0.1 mM reduced the light sum by 1.5- and 2.4-fold, respectively. Further increases in DNICs-GS concentration (0.25 and 0.5 mM) resulted in nearly complete suppression of chemiluminescence (Figure 4a).

Interestingly, high concentrations of GSH (0.25 and 0.5 mM) produced a similar suppressive effect on chemiluminescence (Figure 4c,d). Meanwhile, at 0.05 mM GSH, a stimulatory effect on chemiluminescence was observed in the system with *t*-BOOH and cytochrome *c*. This effect is consistent with previously obtained data for the CumOOH system, where stimulation of chemiluminescence was also observed at low GSH concentrations.

Thus, the studied DNICs exhibit antioxidant properties in model systems containing cytochrome *c* and organic hydroperoxides. The observed differences in the effectiveness of DNICs’ action in systems with CumOOH and *t*-BOOH may be attributed to both the physicochemical properties of the hydroperoxides themselves and the specific features of their interactions with components of the reaction mixture. The following mechanisms can be proposed. First, these differences may be related to the varying degrees of hydrophobicity of the hydroperoxides. CumOOH contains an aromatic cumyl ring, making it more hydrophobic than *t*-BOOH. Consequently, CumOOH may primarily interact with hydrophobic domains of cytochrome *c*, limiting its availability for reactions occurring in the bulk solution. In contrast, *t*-BOOH, which is uniformly distributed throughout the reaction mixture, participates more effectively in reactions with cytochrome *c* and other dissolved components, including DNICs and GSH, resulting in a higher yield of free radical products and more intense and prolonged chemiluminescence. Second, differences in chemiluminescence kinetics in the presence of different hydroperoxides may stem from variations in the rate and mechanism of their decomposition. The compound *t*-BOOH generates more reactive and short-lived radicals that actively participate in chain oxidation processes and efficiently oxidize luminol, leading to strong and sustained chemiluminescence. In contrast, CumOOH produces less reactive but more stable radicals, which may undergo recombination, resulting in lower radical yields and weaker chemiluminescence. Third, the observed differences in hydroperoxide behavior can be explained by their differing sensitivity to DNICs and GSH. In the *t*-BOOH system, where radicals are generated in the bulk solution, DNICs-GS more effectively “trap” them, explaining the extension of the latent period and suppression of luminescence. In the CumOOH system, where radicals are generated locally (near the protein), access of DNICs-GS to them may be limited, resulting in a less pronounced inhibitory effect.

### 2.2. The Effect of DNICs-GS and Free GSH on the Level of Organic Free Radicals in a Reaction System Containing Cytochrome c and tert-Butyl Hydroperoxide

**Figure 5:**
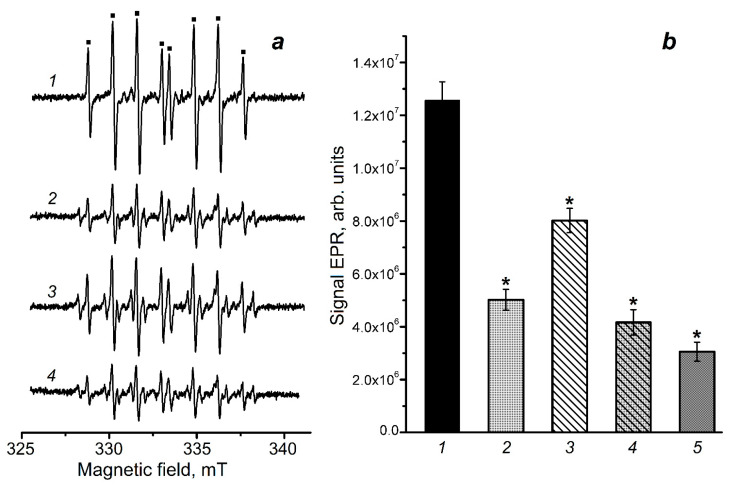
The effect of DNICs-GS and GSH on the content of DEPMPO adducts with organic radicals formed in the cytochrome *c* reaction with *t*-BOOH. The reaction mixture contained 150 mM K,Na-phosphate buffer (pH 7.4), 1 mM ferricytochrome *c*, 4 mM *t*-BOOH, and 75 mM DEPMPO. Panel (**a**) shows electron paramagnetic resonance (EPR) spectra of DEPMPO adducts, the symbol (■) marks lines of *tert*-butoxyl radical adduct (DEPMPO-OR); on panel (**b**) column heights show the amplitude of DEPMPO-OR EPR signal (evaluated on the third line of the spectrum). 1—reaction mixture without admixtures (control), 2—(1) + 0.6 mM DNICs-GS, 3—(1) + 1 mM GSH, 4—(2) + 0.6 mM DNICs-GS + 1.2 mM GSH, 5—(1) + 1.2 mM DNICs-GS. EPR spectra were recorded 4 min after mixing the components (*n* = 4). Groups that are significantly different from the control are marked *.

The production of free radicals during *t*-BOOH interaction with cytochrome *c* was also studied by electron paramagnetic resonance (EPR) spin trap spectroscopy. The 5-(diethoxyphosphoryl)-5-methyl-1-pyrroline-N-oxide (DEPMPO) spin trap was used to detect these free radical intermediates (Figure 5a). Spin adducts of DEPMPO with various free radicals are well-known to have specific EPR spectra [10,44,45]. The reaction mixture contained 1 mM ferricytochrome *c* and 4 mM *t*-BOOH.

Analysis of the EPR spectra presented in Figure 5a indicates that the predominant free radical in this reaction system is the alkoxyl (*tert*-butoxyl) radical, which is formed during homolysis of the peroxide group (Reaction (1)) and recombination of peroxide radicals (Reaction (4)). It can be seen that DNICs-GS decrease the level of organic free radicals formed in the reactions of t-BOOH with cytochrome *c* heme groups. Thus, DNICs-GS at concentrations of 0.6 and 1.2 mM lower the content of alkoxyl radicals in the reaction mixture by 2.4 and 4.2 times, respectively (Figure 5b). In the presence of 1.2 mM GSH, the content of these radicals decreases by 1.6 times. Adding GSH along with DNICs-GS enhanced their effect only to a minor extent (Figure 4, spectrum and column 4). Thus, the antiradical effect of DNICs-GS was significantly higher than that of free GSH at concentrations corresponding to the one of glutathione ligands of the complexes.

As expected, there was no noticeable number of thiyl radicals in experiments with DNICs-GS and GSH. However, EPR spectra characteristic of carbon-centered radicals, such as the methyl radical formed during the decay of the *tert*-butoxyl radical, were observed [10]. It should be noted that thiol radicals can oxidize amino acid residues of proteins and peptides to form carbon-centered radicals [46,47]. Thus, we cannot rule out that in our experiments, thiyl radicals of glutathione, as well as free radical intermediates of Reactions (5)–(7), interact with the protein part of cytochrome *c*.

### 2.3. The Effect of DNICs-GS and GSH on the Formation of Cytochrome c Oligomeric Forms and on 2-deoxy-D-ribose Oxidation

Under the impact of prooxidants, the oxidative modification of proteins, e.g., hemoproteins, leads to degradation of the polypeptide chain or to the cross-linking of protein molecules. This modification may be caused by free radicals, highly oxidized heme groups, and peroxynitrite. In the course of this process, high-molecular-weight oligomeric aggregates are formed [46]. In case of cytochrome *c*, these aggregates arise from its interaction with hydrogen peroxide and cholesterol hydroperoxides, as well as tyrosine phenoxyl radicals, which are involved in forming cross-links [11,17,48].

**Figure 6:**
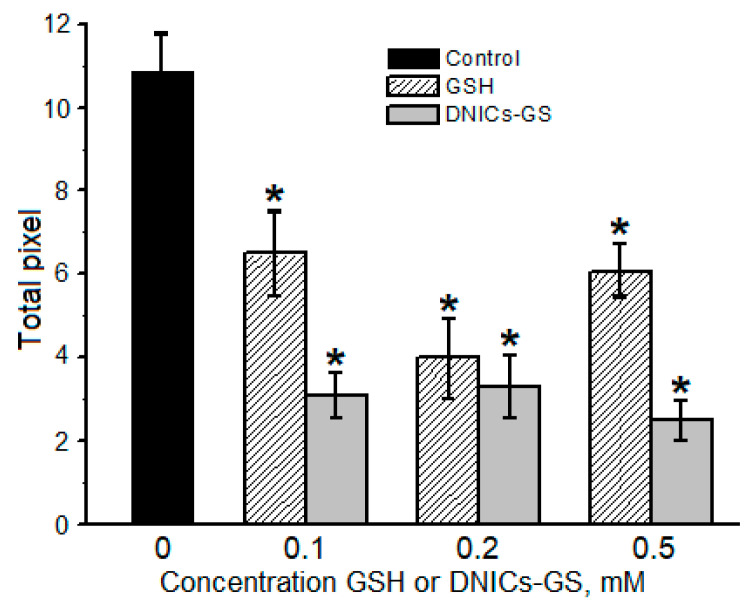
The effect of DNICs-GS and GSH on the formation of oligomeric cytochrome *c* forms in the presence of *tert*-butyl hydroperoxide. The data shown were obtained by processing electrophoretic images using the “Image Lab Software 6.0.1 (Bio-Rad, Hercules, CA, USA)” program. Groups that are significantly different from the control are marked *. The gel image is shown in Supplementary Materials, Figure S1.

By means of SDS-electrophoresis, we showed that *t*-BOOH and CumOOH formed cytochrome *c* oligomers (Figure 6, the gel image is shown in Supplementary Materials). In *t*-BOOH-containing reaction mixture, DNICs-GS decreased the total amount of cytochrome *c* oligomeric aggregates by 3.5 times at 0.1 mM concentration of complexes (Figure 6). Of note, the increase in the DNICs concentration did not significantly change their effect (Figure 6). GSH also inhibited the formation of the cross-linked cytochrome *c* aggregates, with the maximum effect at 0.2 mM concentration. An increasing GSH concentration to 0.5 mM diminished its inhibitory effect.

**Figure 7:**
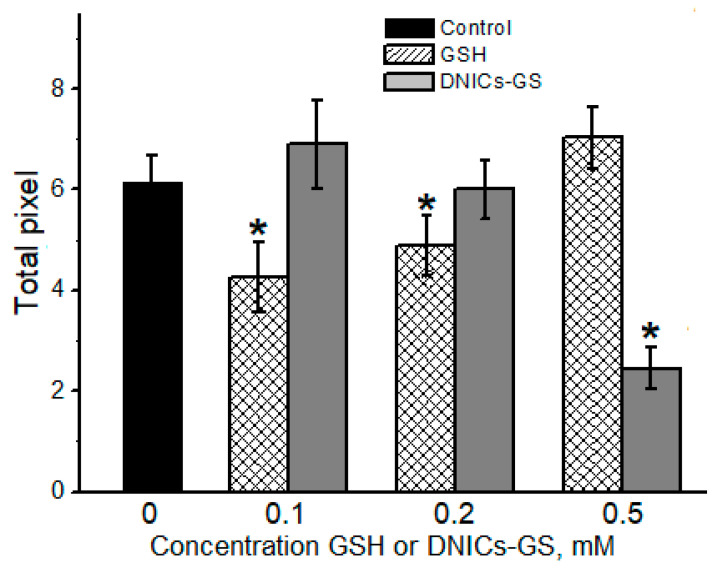
The effect of DNICs-GS and GSH on the formation of oligomeric cytochrome *c* forms in the presence of cumene hydroperoxide. The data shown were obtained by processing electrophoretic images using the “Image Lab Software 6.0.1 (Bio-Rad)” program. Groups that are significantly different from the control are marked *. The gel image is shown in Supplementary Materials, Figure S2.

DNICs-GS inhibited the oxidative modification of cytochrome *c* in the presence of CumOOH by 2.5 times, but did so only at 0.5 mM concentration (Figure 7, the SDS-electrophoresis gel image is shown in Supplementary Materials). By contrast, GSH, at 0.1 mM and 0.2 mM concentrations, slightly decreased the formation of cytochrome *c* oligomers, while at 0.5 mM concentration it was ineffective. The ambiguous effect of GSH on the formation of dityrosine covalent cross-links between cytochrome *c* molecules may be due to the almost equilibrium nature of the reduction reaction of tyrosine phenoxyl radicals by this thiol [49]. Therefore, at high concentrations, glutathione radical (GS^•^) can shift the equilibrium towards the formation of free tyrosine radical.

**Figure 8:**
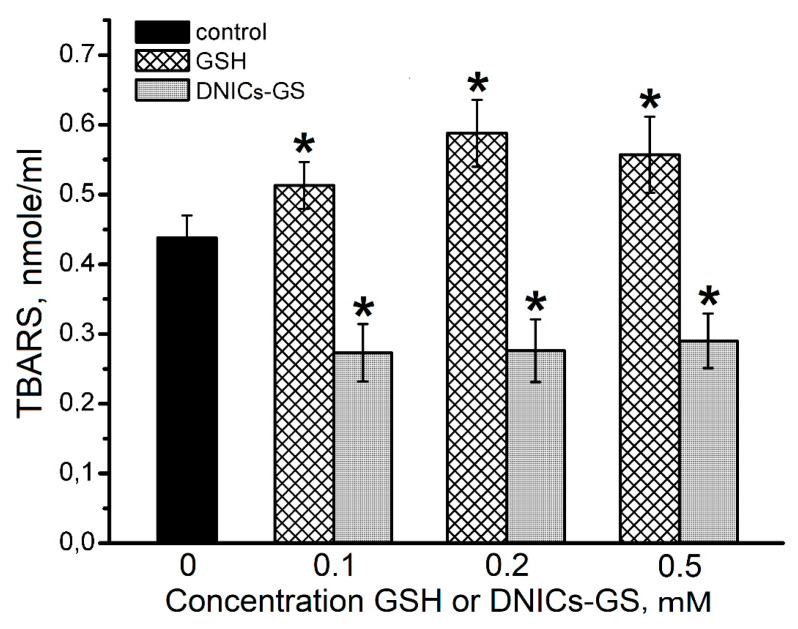
The effect of DNICs-GS and GSH on the formation of TBA-reactive products during 2-deoxy-D-ribose oxidation in cytochrome *c*/*t*-BOOH system. The gray columns represent DNICs-GS, the shaded ones—GSH. Groups that are significantly different from the control are marked *.

The data presented in Figure 8 demonstrate that DNICs-GS decreased TBARS level by approximately 1.5 times at concentrations ranging from 0.1 to 0.5 mM. At the same time, GSH slightly stimulated the oxidative destruction of deoxyribose. The fact that an increase in DNICs-GS concentration did not lead to any sufficient inhibition of oxidative cytochrome *c* modification as well as decreased deoxyribose destruction in the presence of *t*-BOOH (Figure 6 and Figure 8) indicates the balance of both prooxidant and antioxidant processes. It is likely that during the oxidative DNICs-GS destruction GS^−^ and iron ions are released. This further leads to the formation of glutathione-derived radicals, as well as peroxide and alkoxide radicals in Fenton-type Reactions (9) and (10):

Fe^III^ + ROOH → Fe^II^ + ROO^•^ + H^+^ (9)

Fe^II^ + ROOH → Fe^III^ + RO^•^ + OH^−^ (10)

In this regard, it is important to highlight that including iron ions into DNICs inhibits the Fenton-type reactions [50].

When H_2_O_2_ interacts with cytochrome *c* in cells it causes not only the oxidative damage of protein chain, but also the oxidation of other biomolecules. Thus, the oxidation of deoxyribose (2-deoxy-D-ribose) with TBARS (thiobarbituric acid-reactive substances) being formed was shown [48]. In our experiments, oxidative destruction of deoxyribose was observed in the cytochrome *c*/*t*-BOOH system (Figure 8).

### 2.4. DNICs Formation with Participation of Cytochrome c and Nitroxyl Anion

The pool of chelatable or labile redox-active iron in mitochondria plays an important role in DNIC biosynthesis [32,33,34]. Both NO^•^ and the product of its one-electron reduction—the nitroxyl anion NO^−^, as well as the protonated form of the latter—HNO, can take part in DNIC formation [13]. In living systems, NO^•^ and nitroxyl anion convert to one another when they interact with various redox-active compounds, including coenzyme Q, cytochrome *c*, and other components of the mitochondrial respiratory chain [1,18,51,52]. It is assumed that NO^−^/HNO is produced by mitochondrial NO synthase [53].

**Figure 9:**
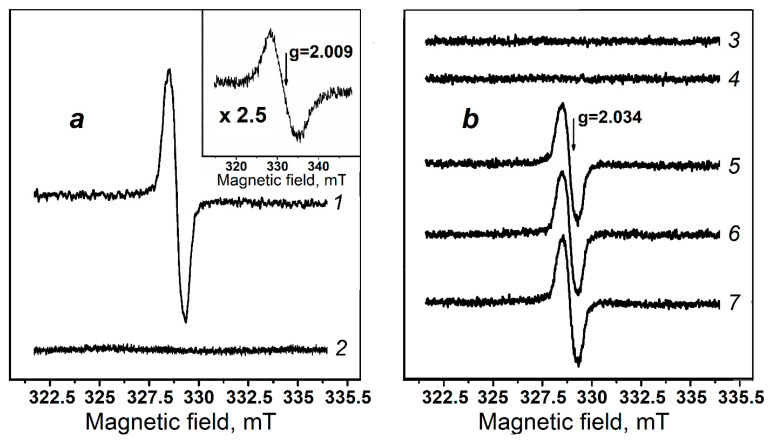
Panel (**a**): EPR spectra of DNICs-GS and nitrosylated cytochrome *c*. EPR spectra of reaction mixtures containing 2.5 mM DNICs-GS and 20 mM GSH (spectrum 1); 10 mM Angeli’s salt, 0.75 mM ferricytochrome *c* (spectrum 2, recorded 8 min after mixing components); Insert: EPR spectrum of cytochrome *c* nitrosylated by heme iron. The reaction mixture contained 2.2 mM ferrocytochrome *c* and 60 mM PAPA/NONOate (the spectrum recorded 16 min after mixing components). Panel (**b**): Formation of DNICs-GS with the participation of Angeli’s salt and ferricytochrome *c*. EPR spectra of reaction mixtures containing 1 mM FeSO_4_, 10 mM Angeli’s salt, 20 mM GSH (spectra 3 and 4, recorded 3 and 8 min after mixing components, respectively); 1 mM FeSO_4_, 10 mM Angeli’s salt, 0.75 mM ferricytochrome *c*, 20 mM GSH (spectra 5, 6 and 7, recorded 3, 8 and 16 min after mixing components, respectively). All samples contained 150 mM K,Na-phosphate buffer (pH 7.4).

Using EPR spectroscopy, we have shown that DNICs-GS are formed in the reaction mixture containing bivalent iron ions, the Angeli’s salt—donor of nitroxyl anion, GSH, and ferricytochrome *c* (Figure 9). During the first three minutes, DNICs-GS level reaches the maximum and then—the plateau (Figure 9, spectra 5–7). Importantly, in the absence of ferricytochrome *c*, DNICs-GS are not formed in this model system. Based on these data, we suppose that the source of NO^•^ for DNICs-GS synthesis is the single-electron oxidation of NO^−^ by ferricytochrome *c* (Reaction (11)):

Cyt-Fe^III^ + NO^−^ → Cyt-Fe^II^ + NO^•^ (11)

Thus, ferricytochrome *c* catalyzes NO^−^ conversion to NO^•^, which further gets incorporated into DNICs. Reaction (11) is reversible, yet the formation of DNICs shifts the equilibrium to NO^•^ production.

Interestingly, in our experimental conditions no significant amount of cytochrome *c* nitrosylated by heme iron was formed (Figure 9, spectrum 2). Additionally, EPR spectroscopy did not detect any nitrosylation of ferricytochrome *c* during incubation with DNICs-GS and DNICs-PO_4_^−^. Nitrosylation of heme iron was observed only when ferrocytochrome *c* was incubated with a high concentration of NO^•^ donor PAPA/NONOate (Figure 9, Inset). It should be noted that the reduction of ferricytochrome to ferroform was induced by sodium dithionite, which also led to a decrease in oxygen content in the reaction mixture. In other experiments, oxygen was supplied to the reaction mixture from ambient air, as EPR spectra were recorded in gas-permeable capillaries. This approach brought the conditions of these measurements closer to those used in chemiluminescence experiments.

## 3. Discussion

It has been previously shown that DNICs play a crucial role in regulating biomolecule peroxidation processes [22,23,25,28,29,33,34]. This regulation is particularly important for mitochondria, being the main sources of ROS in eukaryotic cells [51].

Based on the data obtained, we may infer that the antioxidant effect of the studied DNICs in model systems containing cytochrome *c* is primarily determined by the properties of NO ligands, including their ability to release NO^•^. This assumption is consistent with the fact that antioxidant activity of DNICs-PO_4_^−^ and DNICs-GS in our experiments was comparable (Figure 2). However, contrasted with DNICs-GS, free GSH was less effective as an antioxidant, or even exhibited some prooxidant properties. Indeed, we have previously established that thiol ligands of DNICs are less reactive than free thiols [54]. It is also worth noting that in the used model systems the antioxidant effect of DNICs is most likely unrelated to the nitrosylation of cytochrome *c* heme iron.

It is known that NO^•^ can interact with carbon-centered, alkoxyl, and peroxyl radicals in Reactions (12)–(15) below [14,15,29,55,56]:

R^•^ + NO^•^ → RNO (12)

ROO^•^ + NO^•^ → ROONO (13)

RO^•^ + NO → RONO (14)

ROONO → RO^•^ + ^•^NO_2_ → RONO_2_ (15)

The rate constants of Reactions (12)–(15) are close to diffusion-controlled ones and are 1–3 × 10^9^ M^−1^s^−1^ [15,42,55]. Due to these reactions, NO^•^ prevents the cytotoxic effect of *t*-BOOH and inhibits lipid peroxidation [14,15,42,55].

Meanwhile, DNICs-GS are known to reduce the oxoferryl myoglobin (Mb) and hemoglobin (Hb) forms to non-toxic metforms [25,26]. This process may also depend on NO^•^, which interacts with oxoferryl heme groups in Reaction (16) [26,57]:

porphyrin-Fe^IV^=O + NO^•^ ⟶ porphyrin-Fe^III^-ONO ⟶ porphyrin-Fe^III^ + NO_2_^−^ (16)

The rate constants of Reaction (16) for oxoferryl Mb and Hb forms are 1.8 × 10^7^ M^−1^s^−1^ and 2.4 × 10^7^ M^−1^s^−1^, respectively [57]. It can be assumed that the rate constant of Reaction (15) involving Cyt-Fe^IV^=O has a similar value. Consequently, the rate of Cyt-Fe^IV^=O reduction by NO^•^ is significantly lower than that of the latter’s recombination with organic free radicals (Reactions (12)–(15)). Thus, it is these reactions that determine the antioxidant effect of DNICs-GS and DNICs-PO_4_^−^ detected by chemiluminescence (Figure 2). This assumption is consistent with the results of EPR spectroscopy using the DEPMPO spin trap (Figure 4). However, highly oxidized heme groups play an important role in the cytotoxic effect of *t*-BOOH [58] and contribute to the oxidation of amino acid residues and other biomolecules [6,7,48]. In case of cytochrome *c*, tyrosine phenoxyl radicals and further dityrosine cross-links are formed during the peroxidase cycle [4,6,17,48]. Another mechanism for preventing the appearance of dityrosines and cross-linked cytochrome *c* aggregates is the formation of 3-nitrotyrosine during NO^•^ interaction with tyrosine radical [17]. Meanwhile, iron ions released during heme degradation and catalyzing Fenton-type reactions [48] may contribute to the formation of cytochrome *c* oligomers and in the oxidation of deoxyribose. In the presence of H_2_O_2_, these reactions produce a hydroxyl radical (^•^OH), and in the case of organic hydroperoxides—alkoxyl and peroxyl radicals (Reactions (9) and (10)). In our experiments on the oxidative modification of cytochrome *c*, adding DTPA chelator prevented the effect of iron ions (Figure 5 and Figure 6).

It cannot be ruled out though, that various free radical intermediates, including protein-associated, as well as the highly oxidized forms of cytochrome *c* heme, can directly interact with DNICs NO ligands either. Importantly, the kinetic characteristics of these reactions may significantly differ from reactions with free NO^•^. One may further suggest that the reaction of *t*-BOOH with nitric oxide bound to heme or non-heme iron produces the corresponding alcohol (*t*-BOH) and nitrite (NO_2_^−^) [58]. The differences in the interaction of *t*-BOOH and CumOOH with NO DNICs ligands could account for the differences found in the oxidative modification of cytochrome *c* (Figure 6 and Figure 7).

Apparently, O_2_^•−^ and peroxynitrite directly react with NO DNICs ligands, resulting in the utilization of these prooxidants [22,28,29,33]. Meanwhile, O_2_^•−^ can stimulate the formation of DNICs in mitochondria [33]. Various mechanisms of DNIC formation in cells and organelles are being debated in the pertinent literature [20,22,33,34,59]. This process is assumed to involve iron-sulfur proteins, ferritin, the pool of labile iron, as well as NO^•^ and its metabolites—NO^−^, NO_2_^−^, and S-nitrosothiols. We have shown that NO^−^/HNO oxidation by ferricytochrome *c* may play a significant role in DNIC biosynthesis. Since nitroxyl anion has both prooxidant and antioxidant properties [13,60,61], the utilization of NO^−^/HNO with the formation of DNICs is supposed to affect the balance of these properties in mitochondria.

Nitrosylation of cytochrome *c* by heme iron is known to stimulate apoptosis [62]. DNICs containing thiosulfate or N-ethylthiourea have been shown to exhibit pro-apoptotic effects [23,35]. Still, nitration of cytochrome *c* by Tyr74 residue prevents apoptosis [63].

To sum up, here we have demonstrated that physiological DNIC variants inhibited the prooxidant effect of cytochrome *c* in its interaction with organic hydroperoxides. Therefore, DNICs may affect the initial stage of apoptosis being associated with activating the free radical oxidation in mitochondria.

## 4. Materials and Methods

### 4.1. Materials and Reagents

The following reagents were used in the study: cytochrome *c* from equine heart (ferricytochrome *c*), ferrous sulfate heptahydrate (FeSO_4_ × 7 H_2_O), sodium phosphate dibasic (Na_2_HPO_4_), potassium phosphate monobasic (K_2_HPO_4_), sodium dithionite (Na_2_S_2_O_4_), L-glutathione reduced (GSH), cumene hydroperoxide (CumOOH), *tert*-butyl hydroperoxide (*t*-BOOH), 5-amino-2,3-dihydrophthalazine-1,4-dione (3-aminophthalic hydrazide, luminol), diethylenetriaminepentaacetic acid (DTPA), 2-amino-2-(hydroxymethyl)-1,3-propanediol (Tris), polyacrylamide (PAA), dodecyl sulfate (SDS), glycerol, glycine, Coomassie brilliant blue R-250, bromophenol blue, thiobarbituric acid (TBA), trichloroacetic acid (TCA), 2-deoxy-D-ribose—“Sigma-Aldrich” (St. Louis, MO, USA); 4-hydroxy-(2,2,6,6-tetramethylpiperidin-1-yl)oxyl (4-hydroxy-TEMPO)—“Oxis” (Portland, OR, USA); 5-(diethoxyphosphoryl)-5-methyl-1-pyrroline-N-oxide (DEPMPO), Angeli’s salt (Sodium trioxidinitrate), (Z)-1-[N-(3-aminopropyl)-N-(n-propyl)amino]diazen-1-ium-1,2-diolate (PAPA NONOate)—“Cayman Europe” (Tallinn, Estonia).

The phosphate-bound DNICs (DNICs-PO_4_^−^) were synthesized as described in [29]. Gaseous nitric oxide was passed through FeSO_4_ solution (5.5 mM) in 100 mM K,Na-phosphate buffer (pH 7.0) in the Thunberg tube. Generated DNICs incorporated all the introduced iron. DNICs with glutathione ligands (DNICs-GS) were obtained by adding the GSH solution to phosphate DNICs at a molar ratio of 2:1. This preparation mainly contained binuclear diamagnetic form of DNICs-GS. To obtain a paramagnetic mononuclear form of the complexes, an excess of GSH was added to DNICs-GS preparation. Aliquots of DNICs preparations were stored at liquid nitrogen temperature (−195.75 °C). The DNIC concentration was calculated from the integral intensity of EPR signal of these complexes, using 4-hydroxy-TEMPO spin label as a standard. In this case, prior to EPR spectral acquisition, the equilibrium of DNICs was shifted as far as possible toward the mononuclear paramagnetic form by adding cysteine at a 20:1 molar ratio relative to DNICs.

Angeli’s salt (a nitroxyl anion donor) was dissolved in 10 mM NaOH and stored frozen at –70 °C. The final concentration of Angeli’s salt in the experiments was 100 mM.

### 4.2. Luminol-Dependent Chemiluminescence

The formation of free radical intermediates in the reaction of cytochrome *c* with CumOOH was estimated by chemiluminescence using luminol as an activator. This method is widely used to study the interaction between cytochrome *c* and hydroperoxides, as well as to evaluate the effect of various antioxidants at these conditions [24,25,64]. The reaction mixture contained 0.6 mM cytochrome *c* in 100 mM K,Na-phosphate buffer (pH 7.4), 2 mM luminol, and 0.6 mM CumOOH. Glutathione or DNICs with glutathione and phosphate ligands were added to the reaction mixture at concentrations ranging from 0.05 to 0.5 mM. The reaction was initiated by adding CumOOH. The chemiluminescence was registered on the Lum-5773 chemiluminescence analyzer (“DISoft”, Moscow, Russia). The time between mixing components and the chemiluminescence registration did not exceed 3 s. The kinetics was analyzed with constant stirring and thermostating at 37 °C. To quantify chemiluminescence, we used the intensity of glowing and the light sum over a 2000-s period, which was calculated using the area under the kinetic curve with OriginPro 7.5 software package. The range and sensitivity were 1,000,000 (±100), and the reference light was 1000 units (according to the instrument indications).

### 4.3. Electron Paramagnetic Resonance Spectroscopy

EPR spectra were recorded at ambient temperature (25 °C) using an X-band EPR spectrometer E-109E (“Varian”, Palo Alto, CA, USA). Prior to the assays, the reaction mixture was injected into gas-permeable Teflon capillaries PTFE 22 (“Zeus Industrial Products”, Orangeburg, SC, USA). These capillaries were placed in a quartz tube in the resonator of the spectrometer. The instrument settings were as follows: modulation frequency, 100 kHz; time constant, 0.032; microwave power, 10 mW; microwave frequency, 9.14–9.15 GHz; RF modulation amplitudes, 0.4 mT for DNICs, 0.1 mT for DEPMPO spin adducts and 0.05 mT for 4-hydroxy-TEMPO.

### 4.4. Formation of TBA-Reactive Products During the Oxidation of 2-deoxy-D-ribose

A quantity of 0.1 mM cytochrome *c* was added to 10 mM solution of 2-deoxy-D-ribose in 100 mM K,Na-phosphate buffer (pH 7.4). The oxidation reaction was initiated by adding *t*-BOOH to 0.7 mM concentration. The mixture was incubated for 60 min at 37 °C. Then, TCA was added to final 60 mM concentration, TBA—to final 20 mM concentration, and the samples were incubated for 10 min at 90 °C. After incubation, the samples were cooled in the ice bath and centrifuged at 15,000 rpm for 10 min. The absorbance of TBA-reactive products was measured in the supernatant at 532 nm. Their concentration was calculated with the extinction coefficient (ε = 1.56 × 10^5^ M^−1^cm^−1^).

### 4.5. SDS-Electrophoresis

Electrophoresis was carried out in 17% PAAG blocks (150 × 150 × 1 mm) using the Laemmli method [65]. The reaction mixture contained 0.1 mM cytochrome *c* in 10 mM K,Na-phosphate buffer (pH 7.4), 1 mM DTPA and 0.7 mM *t*-BOOH. The mixture was incubated for 15 min at 25 °C. The sample buffer was prepared using 0.1 M Tris-HCl buffer (pH 6.8) with 4% SDS, 0.2% bromophenol blue, and 20% glycerol. It was added to the protein solution in 1:1 ratio, heated for 5 min at 95 °C, and applied to the gel in 10 μL volumes. A 0.2 M Tris-glycine buffer (pH 8.3) containing 0.1% SDS was used as an electrode buffer. Electrophoresis was performed at 4 °C and I = 50 mA, U = 150 B for 200 min, with “Elf-4” (“DNA-Technology”, Moscow, Russia) used as the power supply. After protein separation was completed, the gel was fixed and stained with a solution of Coomassie Brilliant Blue R-250.

### 4.6. Statistical Analysis

The measurements were performed in at least three replicates for each sample. The statistical data were processed based on 3–4 analytical repetitions. The data are presented as a mean ± standard deviation. Statistical differences were determined by means of a one-way ANOVA analysis followed by *post hoc* Tukey’s multiple comparison test.

## 5. Conclusions

The study explored the effects dinitrosyl iron complexes containing glutathione or phosphate anion may have on the pro-oxidative processes associated with the interaction of cytochrome *c* with organic hydroperoxides. Under these conditions, DNICs decreased the oxidative modification of cytochrome *c* demonstrating antioxidant and antiradical properties. Our findings imply that the antioxidant effect of DNICs on cytochrome *c* largely determines the processes in mitochondria ultimately leading to the programmed cell death.

## Data Availability

The original contributions presented in this study are included in the article/Supplementary Material. Further inquiries can be directed to the corresponding author.

## Supplementary Materials

The following supporting information can be downloaded at: https://www.mdpi.com/article/10.3390/molecules30204110/s1, Figure S1: Formation of cytochrome c oligomers under tert-butyl hydroperoxide oxidation conditions; SDS-PAGE electrophoresis in 15% gel; Figure S2. Formation of cytochrome c oligomers under cumene hydroperoxide oxidation conditions; SDS-PAGE electrophoresis in 15% gel.
