# Supplementary material for: Antioxidant Action of Dinitrosyl Iron Complexes in Model Systems Containing Cytochrome c and Organic Hydroperoxides
Source: Molecules. 2025 Oct 16;30(20):4110. doi: 10.3390/molecules30204110 (PMC12566574; doi:10.3390/molecules30204110)
Supplement: Supplementary file 1 [file molecules-30-04110-s001.zip › molecules-3861375-supplementary.pdf]

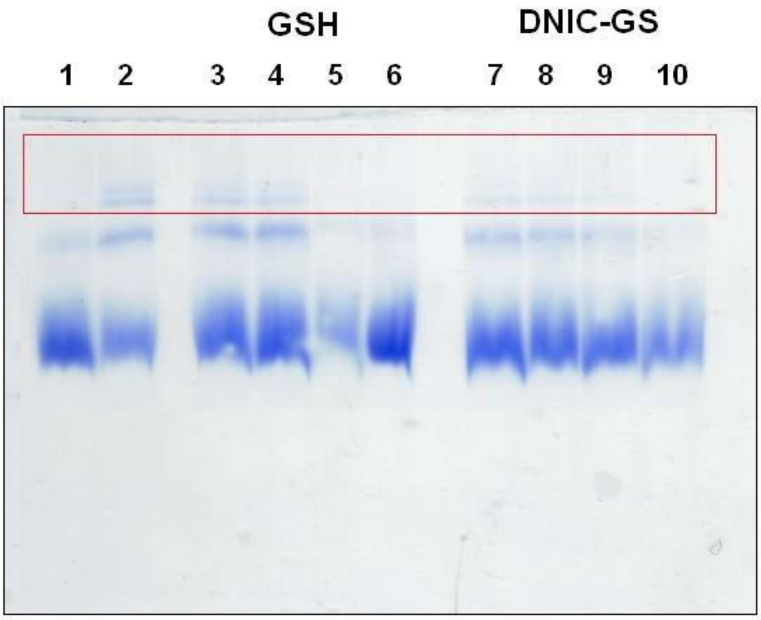

Figure S1. Formation of cytochrome *c* oligomers under *tert*-butyl hydroperoxide oxidation conditions; SDS-PAGE electrophoresis in 15% gel. Gels were stained with Coomassie blue. Lane 1 – control (cytochrome *c*), lane 2 – control (cytochrome *c* + *t*-BOOH). Lanes 3-6: addition of GSH (0.1, 0.2, 0.5, 1 mM). Lanes 7-10: addition of DNICs-GS (0.2, 0.4, 1, 2 mM). The area 0f trimers and higher is highlighted with a red rectangle.

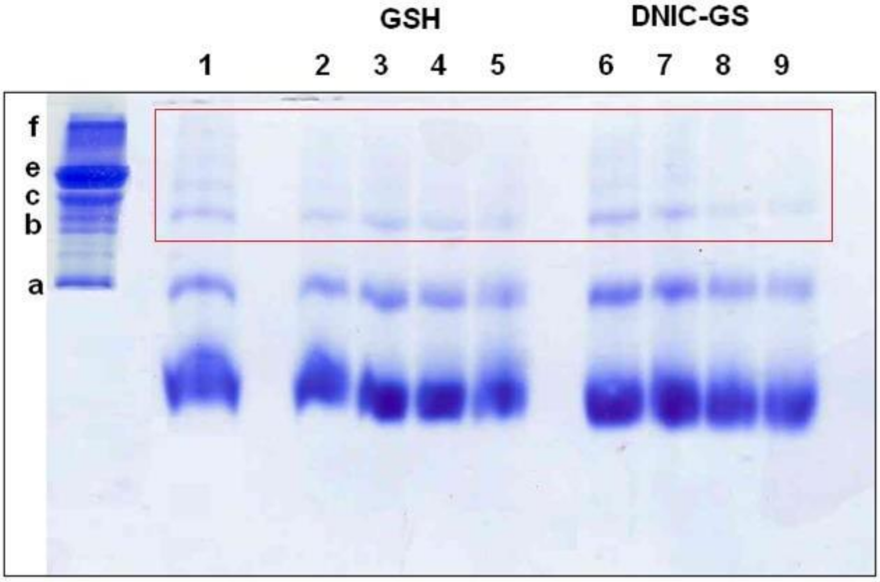

Figure S2. Formation of cytochrome *c* oligomers under cumene hydroperoxide oxidation conditions; SDS-PAGE electrophoresis in 15% gel. Gels were stained with Coomassie blue. Lane 1: control (cytochrome *c* + cumene hydroperoxide). Lanes 2-5: addition of GSH (0.1, 0.2, 0.5, 1 mM). Lanes 6-9: addition of DNICs-GS (0.2, 0.4, 1, 2 mM). The area 0f trimers and higher is highlighted with a red rectangle.

Molecular weight marker array: a – carbonic anhydrase from bovine erythrocytes (Mw 29 kDa), b – albumin from bovine serum (Mw 66 kDa), c – alcohol dehydrogenase from yeast (Mw 150 kDa), e – β-amylase from sweet potato (Mw 200 kDa), f – urease from jack bean (Mw 542 kDa).
